# The RNA Structure of *cis*-acting Translational Elements of the Chloroplast *psbC* mRNA in *Chlamydomonas reinhardtii*

**DOI:** 10.3389/fpls.2016.00828

**Published:** 2016-06-14

**Authors:** Mir Munir A. Rahim, Frederic Vigneault, William Zerges

**Affiliations:** ^1^Department of Microbiology and Immunology, Dalhousie University, HalifaxNS, Canada; ^2^Synthetic Biology Platform, Wyss Institute for Biologically Inspired Engineering, Harvard University, BostonMA, USA; ^3^Biology Department and Centre for Structural and Functional Genomics, Concordia University, MontrealQC, Canada

**Keywords:** photosystem II, secondary structure, stem-loop, dimethyl sulfate, 5′ UTR

## Abstract

Photosystem II is the first of two light-driven oxidoreductase complexes in oxygenic photosynthesis. The biogenesis of photosystem II requires the synthesis of polypeptide subunits encoded by the genomes in the chloroplast and the nucleus. In the chloroplast of the green alga *Chlamydomonas reinhardtii*, the synthesis of each subunit requires interactions between the 5′ UTR of the mRNA encoding it and gene-specific translation factors. Here, we analyze the sequences and structures in the 5′ UTR of the *psbC* mRNA, which are known to be required to promote translation and genetic interaction with *TBC1*, a nuclear gene required specifically for *psbC* translation. Results of enzymatic probing *in vitro* and chemical probing *in vivo* and *in vitro* support three secondary structures and reveal that one participates in a pseudoknot structure. Analyses of the effects of mutations affecting pseudoknot sequences, by structural mapping and thermal gradient gel electrophoresis, reveal that flexibility at the base of the major stem-loop is required for translation and higher order RNA conformation, and suggest that this conformation is stabilized by *TBC1*. This RNA pseudoknot tertiary structure is analogous to the internal ribosome entry sites that promote translation of certain viruses and cellular mRNAs in the nuclear-cytoplasmic systems of eukaryotes.

## Introduction

In the semiautonomous organelles chloroplasts and mitochondria, the translation of mRNAs, encoding polypeptide subunits of the photosynthetic and respiratory complexes, respectively, requires interactions between their 5′ UTRs and organelle gene-specific translation factors encoded by the nuclear genome. For example, in the chloroplast of the unicellular green alga *Chlamydomonas reinhardtii*, translation of the *psbC* mRNA, encoding the CP43 subunit of photosystem II (PSII), is controlled by interactions between its 547 base 5′ UTR and three nuclear genes: *TBC1*, *TBC2*, and *TBC3*. These *trans*-acting functions were identified from *psbC* translation defects in *tbc1* and *tbc2* mutants, and the ability of a suppressor *tbc3* allele to bypasses the requirement for *TBC1* and *TBC1*-interacting sequences in the *psbC* 5′ UTR (Rochaix et al., 1989; Zerges and Rochaix, 1994; Zerges et al., 1997, 2003). These results support a model in which *TBC1* and *TBC3* conjointly interact with sequences in the *psbC* 5′ UTR to activate translation, while *TBC2* appears to operate via a distinct mechanism, since mutant *tbc2* alleles do not show these genetic interactions.

Random and site-directed mutagenesis previously allowed the identification of *cis-*acting sequences in the *psbC* 5′ UTR that are required to drive translation and are involved in the *trans*-interactions with *TBC1*, *TBC2*, and *TBC3* (Rochaix et al., 1989; Zerges and Rochaix, 1994; Zerges et al., 1997, 2003). Translation absolutely requires a 100 nt sequence in the center of the 5′ UTR (223–320) and the translation initiation region, including a predicted Shine–Dalgarno sequence and the initiation codon (which is GUG rather than the common AUG) (Zerges et al., 2003). The requirement for this 100 nt central sequence supports its role in translational activation, contrasting the repressive role of most *cis*-acting translational regulatory sequences in diverse model organisms (Zerges and Hauser, 2009). The 100 nt sequence in this central region is predicted to form a large stem-loop (SL) structure *in silico* (Rochaix et al., 1989). *TBC1*, *TBC2*, and *TBC3* act via sequences in the *psbC* 5′ UTR, either directly or indirectly through unknown translation factors (Zerges and Rochaix, 1994; Zerges et al., 1997, 2003). The *TBC1* interaction involves the 100 nt central region because a spontaneous suppressor point mutation therein, *psbC-F34suI*, restores *psbC* translation in the *tbc1* mutant background, and is predicted to destabilize this SL in simulations using the mfold server (Zuker, 2003). The *TBC1* interaction also involves the 200 nt region (positions 321–529) between the predicted SL and the Shine–Dalgarno initiation region (533–538), based on *TBC1*-independence of the low level of translation of 5′ UTR deletion mutant screen of this region (Zerges et al., 2003). *TBC3* acts via two regions of the *psbC* 5′ UTR: one is within the interval 27–222; located 5′ to the central region with the predicted SL, and the other is in the translation initiation region. Two bulges on the stem of this SL are also required, since a mutant lacking them, *psbC-FuD34*, is completely defective of *psbC* translation. The absence of these bulges is predicted *in silico* to stabilize the SL structure (Rochaix et al., 1989). Together, these genetic results support a model in which complete or partial melting of the SL is required for *psbC* translation, and the *TBC1* and *TBC3* interactions are required therein. There is currently no biochemical evidence of this SL or the effects of the mutations in *psbC-F34suI* and *psbC-FuD34* on the structure of the *psbC* 5′ UTR.

*TBC2* activates translation of the *psbC* mRNA via 5′ UTR sequences in the interval 391–519 (Zerges et al., 2003) and does not show genetic interactions with *TBC1, TBC3* and the predicted SL (as described above for the latter three). Therefore, *TBC2* appears to function in a distinct step in the activation of *psbC* translation from that involving *TBC1, TBC3*, and the predicted SL (Zerges and Hauser, 2009).

Here we characterize the RNA structure of the *psbC* 5′ UTR by enzymatic probing *in vitro* and chemical probing both *in vitro* and *in vivo*. The results support the formation of the central SL structure with several differences from the structure previously described (Rochaix et al., 1989). These included a smaller apical loop, and additional internal loop and bulge in the stem, and the melting of base-pairs at the base of the stem. In addition, we provide evidence for the formation of a pseudoknot tertiary structure involving the base-pairing of the apical loop of this SL structure with a 3′ sequence element. Finally, we extend our results by *in vivo* chemical probing of the *psbC* mRNA for secondary structure and bound factors in wild-type, *tbc1* and *tbc2* mutant strains. This work begins to extend one of the most extensive genetic analyses of translational control to the biochemical level by revealing experimental evidence in support of a *cis*-acting translational element in the *psbC* 5′ UTR.

## Materials and Methods

### Culture Conditions

*Chlamydomonas reinhardtii* strains (Supplementary Table S1) were grown in Tris-acetate-phosphate (TAP) medium (Gorman and Levine, 1965) under a light intensity of 50–75 microeinsteins (μE) m^-2^ s^-1^.

### Dimethyl Sulfate (DMS) Treatments and Analyses

Dimethyl sulfate (DMS) treatments of cells and total RNA samples were performed as described previously (Zaug and Cech, 1995; Higgs et al., 1999). RNA was extracted with Tri-Reagent (Sigma) following the manufacturer’s protocol. Modified A and C residues were revealed by primer extension reactions from the 5′ end-labeled [^32^P] oligonucleotide primers (Supplementary Table S2), which had been purified from a 12% non-denaturing polyacrylamide gel, and hybridized to the *psbC* mRNA in total RNA samples in 0.5 M HEPES pH 7.0, 3.0 M NaCl, 3.0 mM EDTA and extended by Superscript II (Invitrogen). Hybridizations were carried out during 5 min at 85°C and the subsequent 90 min when samples cooled to room temperature. During the reverse transcription reactions, the temperature was increased as follows: 25°C, 10 min; 35°C, 10 min, 45°C, 40 min; 55°C, 10 min; 75°C, 15 min. Products were resolved on 8% (w/v) denaturing polyacrylamide gels and visualized by autoradiography. Positions of pause sites were determined from sequencing ladders derived from each [^32^P]-labeled primer and the *psbC* 5′ UTR in a plasmid vector. The *psbC* 5′ UTR plasmid was constructed by PCR amplification using Primers 1 and 7 (Supplementary Table S2), followed by cloning in pBluescribe at the *Bam*HI and *Sac*I restriction sites (Sambrook and Russell, 2001).

### Generation of RNA Substrates for Enzymatic Probing Reactions and Thermal Gradient Gel Electrophoresis (TGGE)

RNAs were transcribed with T7 RNA polymerase from cloned wild-type and mutant *psbC* 5′ UTRs that had been amplified by PCR by the upstream Primer 1 (Supplementary Table S2) and the downstream Primer 5 (Supplementary Table S2). The T7 promoter was introduced 5′ to the UTR and fragments were cloned into pUC19. Similarly, the RNA corresponding to 225 bp of the 3′ UTR was transcribed from a cloned fragment that had been PCR amplified with upstream Primer 2 (Supplementary Table S2) which also contains the T7 promoter and the downstream Primer 3 (Supplementary Table S2). All DNA fragment inserts were sequenced to exclude the possibility of mutations introduced during PCR. For the enzymatic probing experiments, RNAs were either 5′ end-labelled using T4 polynucleotide kinase (Fermentas) or 3′ end-labeled with T4 phage RNA ligase (Fermentas) and cytidine 3′,5′-(^32^P)bisphosphate (^32^P-pCp, Amersham). For TGGE, RNAs were uniformly labeled during synthesis with [α-^32^P]CTP. Each RNA was purified from an 8% (w/v) denaturing polyacrylamide gel with 8 M urea (Sambrook and Russell, 2001).

A homemade TGGE apparatus was built based on a model described previously (Wartell et al., 1990). The gel had 6% acrylamide/N,N′methylene-bis-acrylamide (60:1) and the running buffer was Tris-borate-EDTA buffer (Sambrook and Russell, 2001). Temperature melting points were measured as previously described by plotting the slope (baseline of each structural states) between two structural transition and identifying the equidistant point (Szewczak et al., 1998).

Digestions with RNase T1 (Fermentas) and RNase V1 (Ambion) were performed with approximately 3 × 10^5^cpm RNA in 10 μl 10 mM Tris [pH 7], 100 mM KCl, 10 mM MgCl_2_ [Ambion] and either 1μg sheared yeast RNA (10 mg/ml, Ambion) and 1 mU of RNase V1 (1 U/μl, Ambion) for 5 min at 24°C, or 4 μg sheared yeast RNA (Sigma Chemicals) and 500 mU of RNase T1 (100 U/μl, Fermentas) for 2 min at 24°C. Reactions were terminated by addition of 20 μl of precipitation/inactivation buffer (Ambion) and incubation at -20°C for at least 1 h. Products (and undigested substrate) were precipitated with ethanol and resolved by electrophoresis on a 6% denaturing polyacrylamide gel and visualized by autoradiography.

As a molecular weight standard, the 100 bp RNA ladder (Fermentas) was labeled with ^32^P using T4 polynucleotide kinase and [γ -^32^P] ATP, and a molecular weight ladder was prepared by heat-treating ^32^P-labeled RNA (10,000 cpm) with 1.0 μg sheared yeast RNA (Sigma–Aldrich) in 5 μl alkaline hydrolysis buffer (50 mM sodium carbonate/sodium bicarbonate [pH 9.2], 1.0 mM EDTA) for 2 min.

## Results

### Chemical and Enzymatic Probing *in vitro* Reveals the Base-pairing Status of Nucleotide Residues in the *psbC* 5′ UTR

**FIGURE 1:**
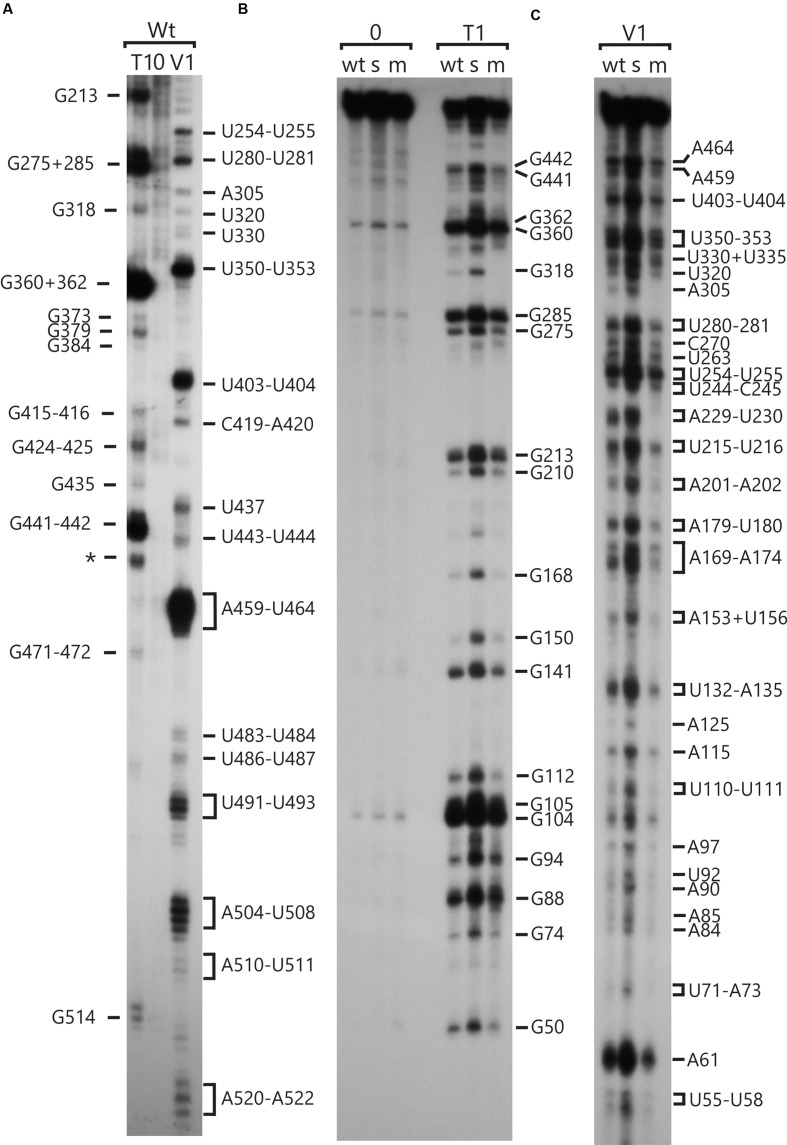
**RNA structure analyses of the *psbC* 5′ UTR by enzymatic probing.** *In vitro* transcribed RNA corresponding to the wild-type and mutant *psbC* 5′ UTR were untreated (0) or digested with RNase T1 (T1) or RNase V1 (V1), which cleave unpaired G residues or RNA helices, respectively. Digestion products were resolved by denaturing PAGE and revealed by autoradiography. The positions of cleavage sites are indicated with respect to their positions on the 5′ UTR, based on the motilities of molecular size markers (not shown). **(A)** The substrate RNA was 3′-^32^P-labeled wild-type *psbC* 5′ UTR. **(B,C)** The substrates were 5′-^32^P-end-labeled RNAs corresponding to the *psb*C 5′ UTR with wild-type sequence (wt), or carrying one of the mutations; *psbC-FuD34* (m), or *psbC-F34suI* (s). The exact position of certain digestion products could not be determined with certainty (^∗^).

**FIGURE 2:**
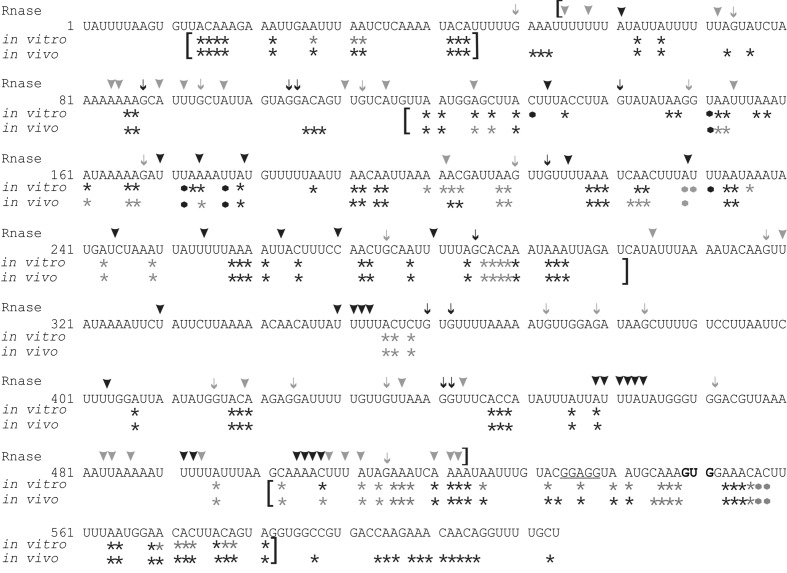
**RNA structure mapping results are shown with respect to the *psbC* 5′ UTR sequence.** Asterisks indicate bases methylated by DMS *in vitro* and *in vivo*. Arrowheads indicate residues that were cleaved by RNase V1. Arrows indicate G residues that were cleaved by RNase T1. Most frequently methylated or cleaved positions are indicated by black asterisks, arrows and arrowheads. Less frequently methylated or cleaved sites are indicated in gray. Brackets indicate results with high accuracy and resolution. The 5′ terminal residue is designated +1, the translation initiation codon (GUG) is in bold, and the predicted Shine–Dalgarno sequence is underlined. Black and gray dots represent strong and weak RT pause sites, respectively.

RNA molecules corresponding to the *psbC* 5′ UTR, with either the wild-type sequence or carrying the mutations *psbC-FuD34* and *psbC-F34suI* (Introduction), were probed *in vitro* for unpaired G residues and base-paired nucleotide residues, using RNase T1 and RNase V1, respectively (Knapp, 1989). Treatment of ^32^P-labeled RNA substrates with each RNase results in site-specific cleavages, which were resolved by denaturing PAGE and revealed by autoradiography (**Figure 1**). In addition, A and C residues of 5′ UTR and beginning of the coding sequence of the endogenous *psbC* mRNA, were probed for base-pairing with DMS *in vitro* in total RNA preparations. A and C residues that are not base-paired are methylated by DMS, while base-pairing prevents their methylation. DMS-methylated A and C residues were revealed by their ability to block reverse transcription (RT) in primer extension reactions from a ^32^P-labeled oligonucleotide primer. Seven primers that hybridize to sites across the 5′ UTR were used for this analysis (Primers 4–10, Supplementary Table S2). RNase cleavage sites and DMS methylated bases were mapped using the length of RNase digestion and primer extension products, which correspond to the distance between them and the ^32^P-labeled end. RNase probing data were obtained between U55 and A523 (**Figure 1**). The mapped sites are shown relative to the sequence in **Figure 2**. RNase V1 cleavage sites were mapped at high resolution at the extremities of this region (A482–A523 and U55–A100) and to within 2–3 nt for the central region (positions G101–A522). RNase T1 cleavages were mapped more precisely because most G residues are sufficiently dispersed in the 5′ UTR to assign each cleavage product to one G. All results were reproducible in at least two independent biological replicate experiments. RNA structures that are supported by these results are presented below, after the description of the DMS mapping results.

DMS accessibility of A and C residues could be obtained for three regions and located in the intervals A14–A44 (**Figure 3A**), A120–A295 (**Figures 3B,C**), and C502–A581 (**Figure 3D**). These regions are bracketed in **Figure 2**. Mapping of DMS accessibility was hampered outside these regions by DMS-independent RT pause sites, which generated primer extension products on the non-DMS-treated RNA (**Figure 4**). These RT pauses probably occur at RNA structures that block the enzyme and are not due to naturally modified bases because the same pattern of stops was observed on an RNA that had been synthesized from a plasmid template *in vitro*. These RT pauses were particularly strong and frequent between positions 369 to 416 (**Figure 4C**) and may reflect a stable RNA structure involving positions within this interval (see below).

**FIGURE 3:**
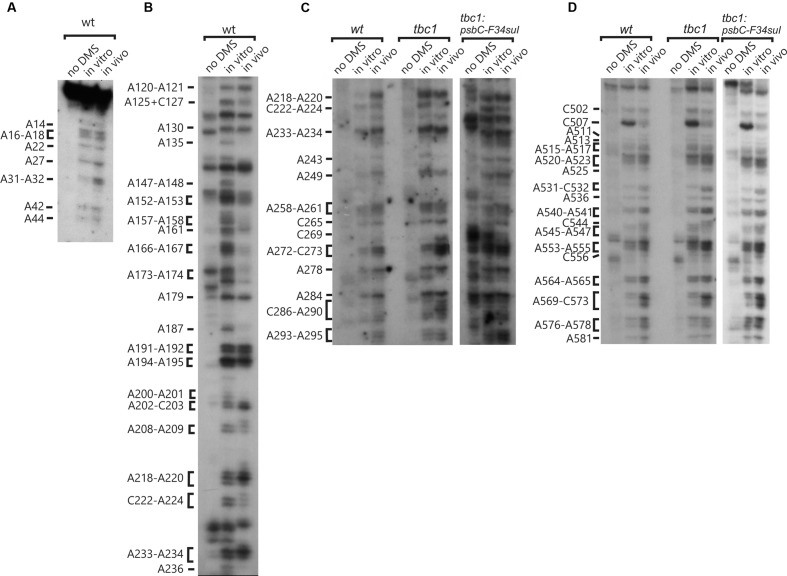
**High accuracy and resolution DMS mapping of A and C residues of the *psbC* 5′ UTR.** DMS-treated (*in vitro* and *in vivo*) and untreated (no DMS) total RNA was analyzed by primer extension reactions using [^32^P] primers specific for the *psbC* 5′ UTR. cDNAs generated by termination of RT at methylated bases were resolved on denaturing PAGE and revealed by autoradiography. **(A)** Methylated A and C residues between positions 14 and 44 of the WT *psbC* 5′ UTR. **(B)** Methylated A and C residues between positions 120 and 236 of the WT *psbC* 5′ UTR. **(C)** Methylated A and C residues between positions 218 and 295 of *psbC* 5′ UTR from the wild-type (WT), the *tbc1* mutant, and *psbC-F34suI*. **(D)** Methylated A and C residues between positions 502 and 581 of *psbC* 5′ UTR from the wild-type (WT), the *tbc1* mutant, and *psbC-F34suI*. Bases methylated by DMS are indicated on the left margin of each autoradiograph.

**FIGURE 4:**
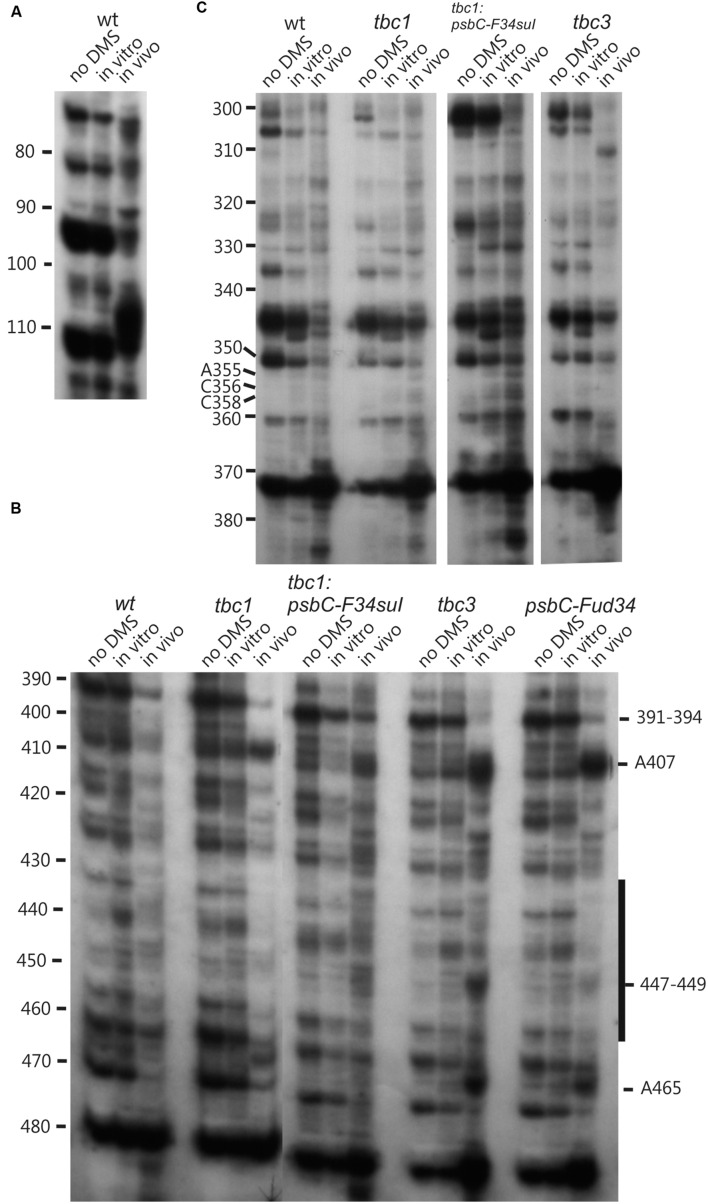
**Lower accuracy DMS mapping of A and C residues of the *psbC* 5′ UTR.** DMS-treated (*in vitro and in vivo*) and untreated (no DMS) total RNA was analyzed by primer extension reactions using [^32^P]-labeled primers specific for the *psbC* 5′ UTR, as described for **Figure 3**. **(A–C)** Regions showing multiple RT stop sites in the untreated as well as DMS-treated RNA from WT and various mutants. Note the strong RT stop sites **(A)**, hypermethylated residues A407 and A465, and hypomethylated residues between positions 430–450 **(C)**, and hypermethylated residues close the position 110 **(A)**.

### Results of Enzymatic and Chemical Mapping Reveal RNA Secondary and Tertiary Structures Formed by the *psbC* 5′ UTR

The A and C residues in the 525–580 interval, including those flanking the GUG initiation codon (549–551), were modified by DMS *in vitro*, indicating that this region is unstructured (**Figure 3D**, summarized in **Figure 2**). This is consistent with a previous report that ribosome subunits are directed to the initiation codon of chloroplast mRNAs by the absence of local secondary structure (Scharff et al., 2011). Although, the A and C residues in the 500–525 interval were modified by DMS *in vitro*, this region was also cleaved by RNase V1 (**Figure 1C**), indicating base-pairing. These seemingly contradictory results could be explained by the concurrent existence of distinct conformations involving these bases. Additional work is required to resolve this issue.

**FIGURE 5:**
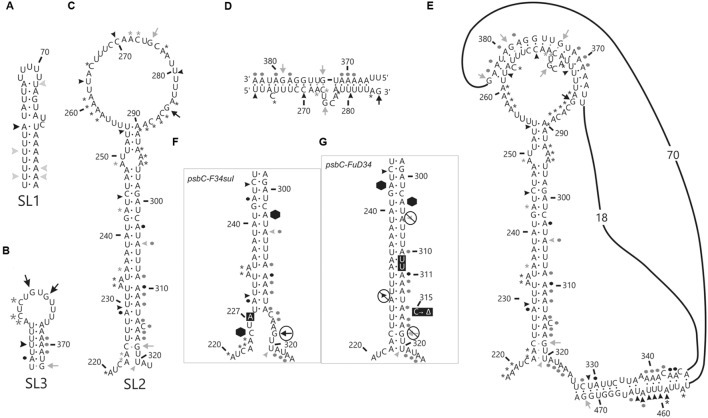
***In vitro* analysis of stem-loop (SL) structures in the *psbC* 5′ UTR.** Asterisks (^∗^) indicate A and C residues that are methylated by DMS *in vitro*. Arrows indicate G residues that are substrates for RNase T1 and arrowheads indicated cleavage sites for RNase V1. Most frequently methylated or cleaved positions are indicated by black asterisks, arrows and arrowheads. Less frequently methylated or cleaved sites are indicated in gray. Hexagons indicate locations of strong premature RT stops. Black dots indicate base-pairs that are strongly supported by the data, while gray dots are partially supported and could be present in a subpopulation of RNA substrate molecules. **(A–C)** DMS methylation and RNase cleavage sites on the three stem-loop structures (SL1, 2 and 3), which were seen in simulations using the *mfold* server (Zuker, 2003). **(D)** Base-pairing of the nucleotides in the loop structure of SL2 to the sequences (367–383) 3′ to the SL2 structure giving rise to **(E)** pseudoknot tertiary structure. **(F,G)** DMS methylation, RNase cleavage and premature RT stop sites on the stem structure of SL2 from *FuD34* and *F34su1* mutant *psbC* 5′ UTR are shown. Note that DMS methylation could not be determined for the *psbC-FuD34* mutant 5′ UTR and the absence of asterisks is due to this, not inaccessibility. **(F,G)** Mutant residues are shown in black boxes with white text.

The *psbC* 5′ UTR forms several SL structures in simulations using the mfold server (Zuker, 2003). These structures are of various sizes and involve localized sequences. Three of these SLs are supported by our data and named SL1, SL2, and SL3 (**Figures 5A–C**). SL1 (54–86) is supported by multiple RNase V1 cleavage sites in the stem; at U55, U58, A61, U72, A84, and A85 (**Figures 1B** and **5A**). The SL3 supported by an RNase V1 cleavage site at U351 in the stem and RNase T1 accessibility of G360 and G362, and DMS-accessibility of A355, C356, and C358 in the apical loop (**Figure 4B**). A weak RNase T1 cleavage site is also present at G373, which is located at the base of the stem suggesting transient melting of its base-pairing with U348 (**Figure 1A**). It should be noted that the nucleotides 368–374 in the stem of SL3 are also predicted to be involved in the formation of a pseudoknot structure described below. SL1 and SL3 were not studied further.

The largest stem-loop structure, SL2, was given particular attention because, as was stated in the Introduction, it is formed by sequences that are required for translation from the *psbC* 5′ UTR and which interact with *TBC1* (Rochaix et al., 1989; Zerges and Rochaix, 1994; Zerges et al., 2003). SL2 is predicted to be formed by sequences in the 220–320 interval, in the center of the *psbC* 5′ UTR (**Figures 2** and **5C**). Results of our DMS methylation and RNase susceptibility analyses provide the first experimental evidence of this structure: (i) The base-pairing of residues in the stem of this structure is supported by strong RNase V1 cleave sites at A229–U230, U244–C245, U254–U255, and a weaker site at A305 (**Figure 1C**). (ii) DMS-accessibility could be determined for 22 of the 35 A and C residues, that are predicted to form the stem structure, due to RT-pause sites in the 300–320 interval (**Figure 4B**). Consequently, the base-pairing status of the predicted bulged residue C315 could not be determined. Nevertheless, the predicted bulge of A233 and A234 from the stem is supported by the DMS-accessibility of these residues (**Figures 3B,C**). (iii) The apical loop of SL2 is supported by DMS-accessibility of A258-261, C265, A272, C273, A278, A284, C286, A287, C288, and A289 (**Figure 3C**), and RNase T1 cleavage sites at G275and G285 (**Figure 1B**).

Our results also reveal that SL2 has features that were not predicted *in silico*, or described previously (Rochaix et al., 1989). Instead of the previous 47 nt apical loop, additional base-pairing was revealed between sequences near the base of the loop; between U251–U254 and A290–A293, such that the apical loop is 35 nt and the apical extremity of the stem has an internal loop composed of A249–U250 and A294–A295 on opposing strands. In addition, four residues that were predicted to be base-paired in the stem showed evidence of transient melting; A223, A224, and A243 were weakly assessable to DMS (**Figure 3C**), and G318 was weakly accessible to cleavage by RNase T1 (**Figure 1**). A223, A224, and G318 are close to the base of the stem where transient melting could be expected. The weak DMS-accessibility of A243 suggests that base-pairing of A243–U244 with U300–A301 transiently melts to create an internal loop (**Figure 5C**).

### A Pseudoknot Tertiary Structure Involves SL2

In the apical loop of SL2 non-paired residues were revealed by strong DMS-methylation sites at A258–A261, C265, A278, A284–A290 and weaker sites at A272 and C273 (**Figure 3C**), by a strong RNase T1 cleavage site at G285, and by a weak site at G275 (**Figure 1**). However, we also found evidence that residues in the apical-most region of the loop are involved in base-pairing, based on strong RNase V1 cleavage sites at U263, C270, and U280–U281 (**Figure 1C**), and the absence of DMS-modification of residues at positions A264, C269, C270, A271, C276, and A277 (**Figure 3C**). Because we could not detect complementarity sequence elements within the apical loop, whose hybridization would explain these base-paired residues, we explored the possibility that the apical loop base-pairs with a complementary sequence in the regions flanking SL2 to form a tertiary structure called a pseudoknot (Draper et al., 1998). When we searched sequences flanking this stem-loop structure for complementarity to the paired sequences in the loop, one such sequence was detected in the interval A367–A385 (**Figure 5D**), beginning 45 nt 3′ to the base of SL2. The data support hybridization of A367–A383 in this interval with U263–U283 in the SL2 apical loop to form the second stem of a pseudoknot (**Figure 5E**). The loop between these two stems would be 45 bases; sufficiently long to extend length of the major stem (28–30 bp). This secondary stem has 16 base-pairs and two bulges (C265 and U274–A277) (**Figure 5D**). Transient melting of the base-pairing between A272–C273 and G373–U374 is supported by weak T1 cleavage and DMS methylation sites (**Figures 1B,C**). The data also support a third stem involving pairing of a sequence located immediately 3′ to SL2 (positions 327–346) with another located 3′ to the secondary stem (positions 455–473) (**Figure 5E**). Finally, this pseudoknot structure is the only one identified by the pseudoknot prediction program KineFold (Xayaphoummine et al., 2005).

### Chemical Probing of A and C Residues *In Vivo* Validates the *In Vitro* Results

Dimethyl sulfate rapidly enters live cells and methylate A and C residues that are neither base-paired nor bound by protein factors (Wells et al., 2000). Therefore, we treated live cells with DMS, prepared total RNA from them, and then mapped DMS methylated A and C residues by RT-mediated primer extension, as was described above for the analyses of DMS-induced methylation *in vitro* (**Figures 3** and **4**, summarized in **Figure 2**). Of the approximately 100 A and C residues that were DMS-accessible *in vitro* in the 548 nt 5′ UTR, only eleven were inaccessible *in vivo* (A135, A147–148, A157–158, A173, A187, A200–201, C222, A236). In addition, DMS-accessibility of C507 was reduced *in vivo*. Only two of these differences involved a residue in SL2 (C222 and A236) and complete consistency was found between the *in vitro* and *in vivo* results supporting second stem of the pseudoknot. C222 was weakly accessible and is part of the base of the stem where transient melting could be expected. A236 is predicted to be paired in the SL2 and, therefore, its DMS inaccessibility *in vivo* provides further experimental support of this structure. The eleven residues accessible only *in vitro* might, *in vivo*, be bound by protein factors or paired in RNA structures that require conditions that were not present in our *in vitro* experiments. Together, the chemical and enzymatic probing data provide evidences for many residues within the *psbC* 5′ UTR that are base-paired and, therefore, involved in the formation of secondary and tertiary structures. The general correspondence of the *in vitro* and *in vivo* mapping results provide confidence that our *in vitro* mapping experiments were performed on RNA structures that are relevant *in vivo*.

A few bases were accessible to both RNase V1 and DMS (e.g., residues at positions 125, 174, 420, 459, 457, see **Figure 2**). This seemingly contradictory evidence for paired and non-paired status, respectively, could represent the concurrent existence of alternative structural features, which we have not identified in these analyses.

### The *psbC-F34suI* Mutation Destabilizes the Major Stem-Loop Structure SL2

In order to determine whether the *psbC-F34suI* suppressor mutation alleviates the requirement for the *trans*-acting *TBC1* function by altering the structure of SL2 (see Introduction), we characterized the structure of this mutant 5′ UTR by enzymatic and DMS probing experiments, as described above for the wild-type 5′ UTR (**Figures 1**, **3**, **4**, and summarized in **5F**). This mutation corresponds to a U to A transition at position 227 in the 3′ strand of the stem and near the base of SL2 (**Figure 5F**). Because this mutation reduces the complementarity between the strands by one base-pair, it was predicted to destabilize the stem (Rochaix et al., 1989). Indeed, relative to the results with the wild-type 5′ UTR, we detected enhanced cleavage by RNase T1 at G318 (**Figures 1B** and **5F**) supporting the enhanced melting of the proximal-most base-pairs of SL2. This mutation also restored *in vivo* DMS accessibility of C222–A224 and of the residues between positions 430 and 460, which showed reduced methylation in *tbc1* mutant (described below), to wild-type levels (**Figures 3C** and **4D**). In addition, *psbC-F34suI* generates strong RT pause sites within the stem (A303) and the apical loop (∼C270) providing further support that it alters SL2 structure (**Figures 3C** and **4B**).

### The *psbC-FuD34* Mutation Stabilizes the Major Stem-Loop Structure SL2

**FIGURE 6:**
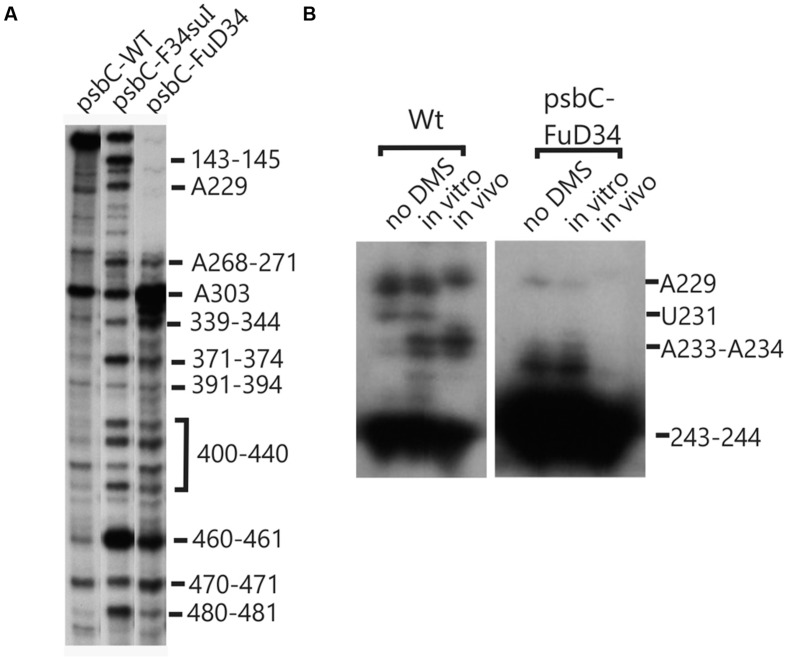
**Structural analyses of the *FuD34 and F34su1* mutant *psbC* 5′ UTRs.** Structure of mutant *FuD34* and *F34su1psbC* 5′ UTRs were probed by primer extension reactions without **(A)** of with DMS treatment **(B)**. Note the strong RT stop sites in *FuD34* and *F34su1* mutant *psbC* 5′ UTRs without DMS-treatment, at positions 229, 241–245, and 301–305, which correspond with each strands of the central stem-loop. A more proximal primer (Primer 8, hybridizing 279–263) was used in **(B)** compared with the more distal primer (Primer 5, hybridizes (549–532) in **(A)**, in order to pass the 301–305 block in *FuD34*.

The *psbC* 5′ UTR mutation, *psbC-Fud34*, abolishes translation of the *psbC* mRNA and was predicted to stabilize SL2 (Rochaix et al., 1989) by removing the two bulges in the stem. The A233–A234 bulge is eliminated by base-pairing of these residues with two inserted U residues, between A309 and A310 and the C315 bulge is eliminated by the deletion of this position (**Figure 5G**). Results of *in vitro* enzymatic probing experiments on an RNA corresponding to the *psbC-Fud34* 5′ UTR revealed the evidence for the stabilization of the base of SL2 as the absence of the RNase T1 cleavages at G318 (**Figure 1B**). This mutant 5′ UTR also lacked two of the three RNase V1 cleavage sites in the SL2 stem; at A229–U230 and A305 (**Figure 1C**). While this could be interpreted as partial melting of the SL2 stem in this mutant, the preponderance of evidence suggests that a higher order structure involving SL2 sterically blocks access of RNase V1 to these base-paired sequences. This evidence includes the results of enzymatic and chemical probing experiments supporting SL2 formation and the enhanced complementarity of the strands of the stem in the *psbC-Fud34* 5′ UTR, meaning that SL2 is more thermodynamically stable than in *wild-type*. On the *psbC-FuD34* mutant 5′ UTR, we detected striking blocks to primer extension by reverse transcription on each strand of the central stem-loop; at 241–245 and 301–305, which, on the wild-type 5′ UTR, were detected as weaker RT pause sites (**Figure 6**). These blocks were so potent that most RT reactions stopped at them and they precluded determinations of base-pairing status based on DMS accessibility. Nevertheless, these potent RT-pause sites, and TGGE results presented in the next subsection, provide additional support of a highly stabilized structure of SL2 relative to the wild-type (Ehresmann et al., 1987). We also detected alteration of DMS accessibility downstream of SL2, which were also detected in *tbc* mutants, described later. These alterations included hypermethylation of A407 and A465, and hypomethylation of residues between positions 430 and 460 (**Figure 4C**).

**FIGURE 7:**
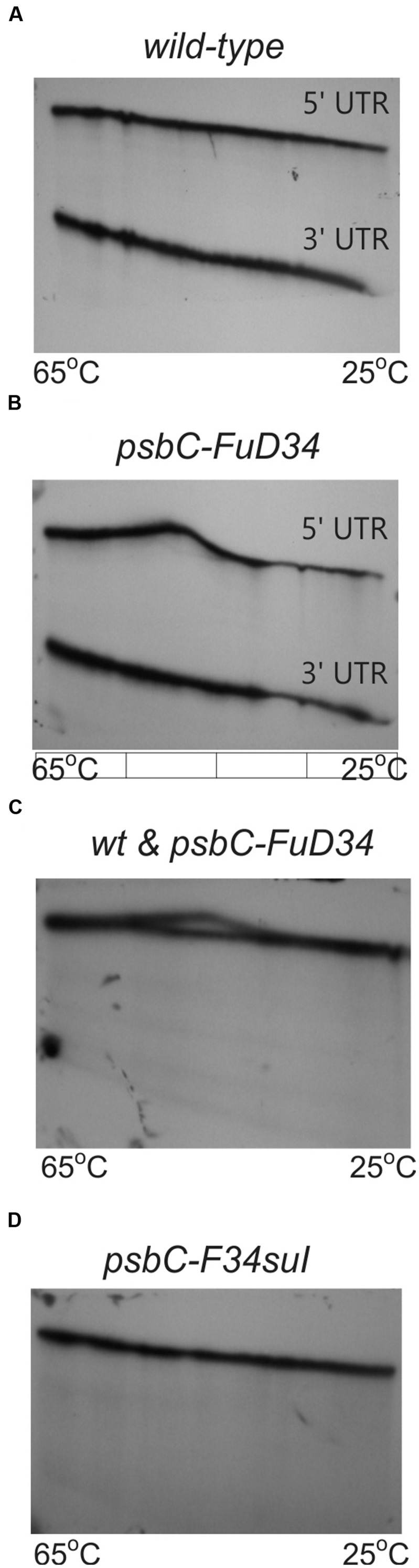
**TGGE melting profile of the *FuD34 and F34su1* mutant *psbC* 5′ UTRs.** TGGE analysis of *psbC* 5′ UTRs from WT **(A)**, *FuD34* **(B,C)** and *F34su1* **(D)** mutants. The *psbC* 3′ UTR RNA was used as control. Note the alteration of electrophoretic mobility in the mutant *FuD34* compared to the WT *psbC* 5′ UTR, while the *F34suI* mutation does not appear to alter the TGGE profile.

TGGE profiles can complement RNA structure probing by chemicals and ribonucleases (Szewczak et al., 1998), and has been used to study the structure of the 5′ UTR of a chloroplast RNA (Klaff et al., 1997). In this technique, a homogeneous sample of an RNA molecule is incubated across a linear temperature gradient from 65 to 25°C. The sample is incubated in a single long well, across the origin of a polyacrylamide gel which has this temperature gradient oriented perpendicular to the direction of electrophoresis. In this temperature gradient, the RNA forms a gradient of structures, ranging from unfolded at the 65°C extreme, to folded at the 25°C extreme, with folding intermediates in the interim. During subsequent electrophoresis, while the temperature gradient maintains the RNA in its adopted conformation, the electrophoretic mobility reveals the degree of structure, which is detected as retarded mobility. As seen in **Figure 7A**, the wild-type *psbC* 5′ UTR showed a linear structural transition in TGGE analysis. In contrast, the *psbC-FuD34* mutant 5′ UTR exhibited a dramatically altered melting profile (**Figures 7B,C**). The conformation(s) adopted by this mutant UTR between 50 and 55°C are less hydrodynamic, and show retarded mobility. Retarded electrophoretic mobility is especially pronounced for RNA molecules with internal loops, such as a spliced intron lariat (Domdey et al., 1984). *In silico* simulations using the mfold server (Zuker, 2003) show SL2 of the wild-type and *psbC-F34suI* mutant 5′ UTRs unfold by melting of the base of the stem beginning at 48°C and 43°C, respectively. By contrast, the SL2 with the *psbC-FuD34* mutation is predicted to unfold by melting of the stem beginning at the apical loop and at 54°C. The *psbC-FuD34* 5′ UTR probably has retarded mobility at 50–55°C because it has an enlarged apical loop, which is not the case for the melting wild-type and *psbC-F34suI* 5′ UTRs (**Figure 7D**). At the 25°C temperature extreme, the wild-type and *psbC-FuD34* 5′ UTRs do not detectably differ in electrophoretic mobility and therefore, within the limits of detection of TGGE, do not differ in structure. Therefore, these results provide further evidence that the SL2 structure in the *psbC-FuD34* 5′ UTR is stabilized, suggesting this may be the basis of its inability to promote translation.

### DMS Probing of the *psbC* mRNA in the *tbc2* Mutant Shows a Footprint on the Coding Region, Which Was Not Detected in Wild-Type, or the *tbc1* or *tbc3* Mutants

**FIGURE 8:**
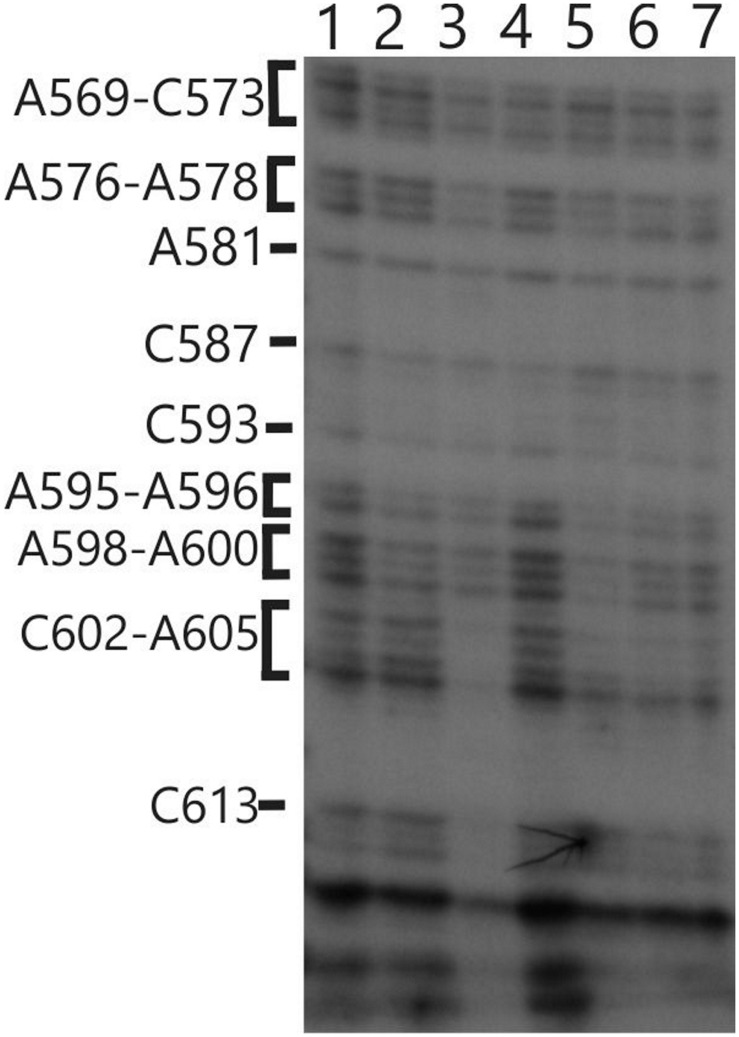
***In vivo* DMS mapping of the 5′ region of the coding sequence from WT and various mutants.** Hypomethylated bases were detected downstream of position 602 on the *psbC* 5′ UTR in the *tbc2* mutant (lane 3). Lane (1) wild-type, Lane (2) *tbc1* mutant, Lane (3) *tbc2* mutant, Lane (4) the *psbC-F34suI* suppressor mutation, Lane (5) tbc3 mutant, Lane (6) *FuD34* mutant, Lane (7) double mutant for *tbc3* and *psbC-FuD34*.

We also used *in vivo* DMS probing experiments to characterize effects of mutations at the *TBC1*, *TBC2* and *TBC3* loci on the *in vivo* status of A and C residues with respect to base-pairing and bound protein. The mutant *tbc2* strain had no reproducible alterations of the DMS methylation pattern relative to the wild-type 5′ UTR. However, drastically reduced DMS accessibility in the *tbc2* mutant was detected in the coding sequence in the interval 600–620, which is downstream of the initiation codon (549–551) (**Figure 8**). This is probably the footprint of a bound protein factor because it is too short to represent a stalled ribosome, which should be at least 30 nt (Zoschke et al., 2013) and it is unlikely to represent an RNA structure, based on *in silico* predictions (with the program mfold) and the DMS-accessibility of the same A and C residues in the wild-type and *psbC-F34suI* mRNAs, both *in vitro* and *in vivo* (**Figures 3D** and **8**). This could be the footprint of proteins that were previously detected by UV-crosslinking in extracts of non-membrane proteins from the *tbc2* mutant, but not in equivalent extracts of wild-type and *tbc1* mutant strains (Zerges and Rochaix, 1994).

### The DMS Methylation Pattern of the 5′ UTR is Altered in the *tbc1* and *tbc3* Mutants

Enhanced DMS accessibility was detected at many of A and C residues upstream from SL2 (**Supplementary Figure S1**). Alterations of the DMS methylation pattern were observed dispersed across the 5′ UTR of the wild-type *psbC* mRNA in the *tbc1* mutant, relative to the wild-type strain for *TBC1* (**Figures 3** and **4**). Within SL2, several alterations were also detected. We detected enhanced *in vivo* DMS accessibility of C269, A272, and C273 within the apical loop (**Figure 3C**) and a reduced DMS accessibility of C222–A224, and A243. Downstream of SL2, a cluster of reduced DMS-accessible residues between positions 426 and 460 was detected (**Figure 4C**). Lastly, C544, located close to the initiation codon, showed enhanced *in vivo* DMS accessibility in the *tbc1* mutant, relative to the wild-type strain (**Figure 3D**). These residues could be involved in the trans-acting translational activation by *TBC1*.

As another criterion for residues in the *psbC* 5′ UTR that are involved in the *trans*-acting translational activation by *TBC1*, we asked whether any of the residues that showed altered DMS-accessibility in the *tbc1* mutant were reversed to the wild-type accessibility by the *psbC-F34suI* suppressor mutation in SL2. When we examined the DMS methylation pattern in the suppressed double mutant strain (*tbc1* mutant and *psbC-F34suI*) certain effects of the *tbc1* mutant on DMS accessibility of the wild-type *psbC* 5 UTR were reversed, while others were not. For example, reduced DMS-accessibility of residues in the 426–489 interval (**Figure 4C**) were restored to the higher accessibility seen in wild-type. The enhanced DMS-accessibility in the stem of SL2 (C269, A271, A272, and C273) also appeared to be restored to the accessibility in wild-type, however, *psbC-F34suI* also resulted in a strong RT pauses which obscured these results (**Figures 3C and 6A**). Finally, the enhanced DMS accessibility of the C544, near the initiation codon, in the tbc1 mutant was also reversed to the wild-type level, suggesting a role of this residue mediating the transacting *TBC1* function (**Figure 3D**). Additional work is required to understand the structural and mechanistic roles of these residues in mediating translational activation by *TBC1*. In SL2, near the base, we found enhanced DMS-accessibility of residues A309–312 in the *tbc3* mutant, suggesting that an internal loop is present in the stem at this position in this mutant which is not present in the wild-type or other mutant stains (**Figure 4B**). In a region that is not required for translation (Zerges and Rochaix, 1994; Zerges et al., 1997, 2003), located between the pseudoknot and the GUG initiation codon, we observed enhanced DMS accessibility in mutant(s) relative to in wild-type. These include A407 and A465 in the *tbc1* and *tbc3* mutants, and A449–C450 in the *tbc3* mutant (**Figure 4C**). Additional work is required to understand the significance of these results with regards to RNA structure or bound trans-acting factors.

## Discussion

This work extends one of the most extensive genetic analyses of translational control in any system to the biochemical level by revealing experimental evidence for a *cis*-acting translational element in the *psbC* 5′ UTR, named here SL2. We also provide evidence that the apical loop of SL2 hybridizes with a downstream sequence located between it and the GUG initiation codon, to form a pseudoknot. The translational activator function of SL2 was demonstrated previously by effects of site-directed mutagenesis on expression of a chimeric reporter transcript in chloroplast transformants (Zerges et al., 2003).

The SL2-containing pseudoknot structure has features of internal ribosome entry sites (IRESs). IRESs in certain eukaryotic viral and a minority of cellular mRNAs, promote 5′-methylguanosine cap-independent translation initiation (Lozano and Martínez-Salas, 2015). IRES elements have diverse structures and promote translation initiation by different mechanisms and some form pseudoknot tertiary structures (Costantino et al., 2008). Involvement of pseudoknot structures in the regulation of translation initiation has also been demonstrated in bacteria (Brantl, 2007; Haentjens-Sitri et al., 2008; Babitzke et al., 2009; Wu et al., 2014). IRES elements have not been found in bacterial genetic systems, although eukaryotic viral IRES can function in bacteria (Colussi et al., 2015). Therefore, our results raise the possibility of a natural IRES-like structure in a “bacterial” genetic system, considering that chloroplast genetic systems are essentially bacterial in most regards.

SL3 and the pseudoknot (**Figures 5B,E**) appear to be mutually incompatible structures because each requires base-pairing interactions by the sequence interval 365–373 (**Figures 5B,D,E**). This could mean that one or the other of these *in silico* predicted structures does not form. Alternatively, SL3 and the pseudoknot could be alternative structures, with populations of each existing concurrently. It is also possible that SL2 and SL3 could, together, be involved in a RNA triple helix. RNA pseudoknot triple helix structures have been described recently (Cash et al., 2013) and involve low complexity sequences (e.g., A and U homopolymers in our case). RNA triple helices are involved in various biological process including translational regulation (Conrad, 2014).

The translation defects in the mutants lacking SL2 sequences were not due to the removal of one or more essential translational elements because they can be reversed by suppressor mutations in *tbc3* or SL2 (*psbC-F34suI*). A potential inhibitory function of the SL2 pseudoknot is revealed by the effect of SL2 structure bearing the *psbC-FuD34* mutation, which is defective in translation (Rochaix et al., 1989). These results are consistent with a regulatory function of the SL2-pseudoknot as a translational switch.

Flexibility at the base of SL2 is critical for its translational activation function based on the following. The *psbC-FuD34* mutation stabilizes the base of SL2 stem, as revealed by results of chemical and enzymatic probing (**Figure 5G**), the potent blocks to RT-mediated primer extension at the mutant SL2 (**Figure 6**), and results of TGGE (**Figures 7B,C**). This mutation also appears to stabilize a higher-order structure which blocks the access of RNase V1 to SL2 stem (**Figures 5C,G**).

Our results suggest that *TBC1* promotes an RNA conformation that requires flexibility of SL2 with respect to the flanking sequences because the *psbC-F34suI* mutation abolishes base-pairing in this region (**Figure 5F**). This RNA conformation could be the SL2-containing pseudoknot because *TBC1* was shown to functionally interact with SL2 and sequences including the 3′ strand of the second stem (interval 320–390) (Zerges et al., 2003). A few other observations suggest that the many dispersed newly methylated sites detected upstream of SL2 in the *tbc1* mutant are due to an effect of *TBC3*. First, none of these methylations are restored by *the psbC-F34suI* suppressor mutation (**Supplementary Figure S1**). Second, the region upstream SL2 was previously shown to be required for the interaction with *TBC3*, but not with *TBC1* (Zerges and Rochaix, 1994; Zerges et al., 1997, 2003). Third, many of these methylations in the *tbc1* mutant are shared with the *tbc3* mutant. Thus, in the *tbc1* mutant, a hypothetical *TBC3*-dependent translational factor might be prevented from binding to this region of the leader, or it might bind constitutively.

Finally, we found in the *tbc2* mutant that a region in the beginning of the coding region is protected from *in vivo* methylation by DMS. These results suggest that the translational block in this mutant is associated with one or more stalled ribosomes or a bound protein factor in this region, which generate this footprint. These sequences, however, are not required for trans-acting *TBC2* function because it can be conferred to translation of a heterologous reporter gene (Zerges and Rochaix, 1994; Zerges et al., 1997). If this footprint is relevant to the translational block in the tbc2 mutant, then it would have to result somehow from effects of *TBC2* on sequences in the 5′ UTR.

## Author Contributions

MR and FV performed the research. MR, FV, and WZ designed the research, analyzed the data and wrote the paper. WZ managed the project.

## Conflict of Interest Statement

The authors declare that the research was conducted in the absence of any commercial or financial relationships that could be construed as a potential conflict of interest.

The reviewer KB and handling Editor declared their shared affiliation, and the handling Editor states that the process nevertheless met the standards of a fair and objective review.

## Supplementary Material

**FIGURE S1 | Dimethyl sulfate (DMS) mapping of A and C residues of the *psbC* 5′ UTR.** DMS-treated (*in vitro and in vivo*) and untreated (no DMS) total RNA was analyzed by primer extension reactions using [^32^P] primers specific for the *psbC* 5′ UTR. cDNAs generated by termination of RT at methylated bases were resolved on denaturing PAGE and revealed by autoradiography. Methylated A and C residues between positions 140 and 195 of *psbC* 5′ UTR from the wild-type (WT), mutants *tbc3* and *tbc1*:*psbC-F34suI*.
