# Supplementary figures and images for: The RNA Structure of cis-acting Translational Elements of the Chloroplast psbC mRNA in Chlamydomonas reinhardtii
Source: Front Plant Sci. 2016 Jun 14;7:828. doi: 10.3389/fpls.2016.00828 (PMC4906055; doi:10.3389/fpls.2016.00828)

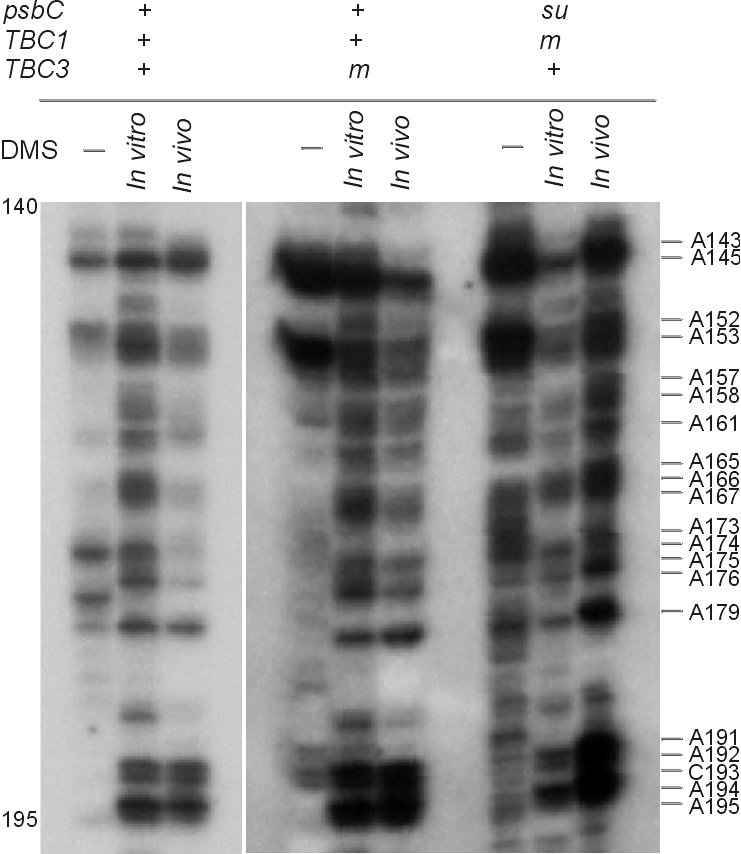

Supplement: Supplementary file 1 [file Image_1.TIF]
