# Supplementary material for: The RNA Structure of cis-acting Translational Elements of the Chloroplast psbC mRNA in Chlamydomonas reinhardtii
Source: Front Plant Sci. 2016 Jun 14;7:828. doi: 10.3389/fpls.2016.00828 (PMC4906055; doi:10.3389/fpls.2016.00828)
Supplement: Supplementary file 2 [file Table_1.DOCX]

**Supplementary Table 1.** *C. reinhardtii* strains (Rochaix *et al*., 1989; Zerges *et al*., 1997).

| Strain | Phenotype | Genotype | |
| --- | --- | --- | --- |
|  |  | Nuclear | Chloroplast |
| *JVD.4A+* | Wild type (WT) | *wild-type* | *wild-type* |
| *F34.3+* | PSII deficient | *tbc1-F34* | *wild-type* |
| *F64+* | PSII deficient | *tbc2-F64* | *wild-type* |
| *RB1.10A* | Partially PSII deficient | *tbc3-rb1* | *wild-type* |
| *FuD34.3* | PSII deficient | *wild-type* | *psbC-FuD34* |
| *F34suI* | Partially PSII deficient | *tbc1-F34* | *psbC-F34suI* |
| *RB1.4A* | Partially PSII deficient | *tbc3-rb1* | *psbC-FuD34* |
