# Supplementary material for: The RNA Structure of cis-acting Translational Elements of the Chloroplast psbC mRNA in Chlamydomonas reinhardtii
Source: Front Plant Sci. 2016 Jun 14;7:828. doi: 10.3389/fpls.2016.00828 (PMC4906055; doi:10.3389/fpls.2016.00828)
Supplement: Supplementary file 3 [file Table_2.DOCX]

**Supplementary Table 2:** Oligonucleotide Primers.

| Primer Name | Primer Sequence^1^ | Position^2^ |
| --- | --- | --- |
| Primer 1 | 5’*GGATCCG*TAATACGACTCACTATAGGGATTTTAAGTGTTACA | 1–16 |
| Primer 2 | 5’GTAATACGACTCACTATAGGGTCATGGTTAGCTTGTTCG | 2007–1989 |
| Primer 3 | 5’-GGATCCTAGTAAATAACAAATCTG | 1780–1804 |
| Primer 4 | 5' TAGCACCTAAAAGTTTACCT | 666–648 |
| Primer 5 | 5’ *ACGAGCCATGGA*CACTTTGCATTACCTCCG | 549–532 |
| Primer 6 | 5' ACCTTTAACAACAAAAATC | 442–424 |
| Primer 7 | 5’ *GAGCT*CATTTTTAAAACACAGAGT | 373–354 |
| Primer 8 | 5' AATTGCAGTTGGAAAGT | 279–263 |
| Primer 9 | 5' ACTTAATCGTTTTTAATTGTT | 210–190 |
| Primer 10 | 5’ CCATTAACATGACAACTGTCCTACTAAT | 123–96 |

^1^ The *psbC* sequences are shown in plain text. Restriction sites are in italics. The T7 promoter is underlined.

^2^ Positions are provided relative to the *psbC* 5' leader terminus. Note that 5' UTR is position 1-546, GTG start codon is position 547-549, *psbC* coding sequene is position 547-1932, and 3' UTR is position 1933-2007
